# Salt-wasting congenital adrenal hyperplasia phenotype as a result of the TNXA/TNXB chimera 1 (CAH-X CH-1) and the pathogenic IVS2-13A/C > G in *CYP21A2* gene

**DOI:** 10.1007/s42000-022-00410-w

**Published:** 2022-10-20

**Authors:** Pavlos Fanis, Nicos Skordis, Leonidas A. Phylactou, Vassos Neocleous

**Affiliations:** 1 Department of Molecular Genetics, Function and Therapy, The Cyprus Institute of Neurology and Genetics, Nicosia, Cyprus; 2 Division of Pediatric Endocrinology, Paedi Center for Specialized Pediatrics, Nicosia, Cyprus; 3 Medical School, University of Nicosia, Nicosia, Cyprus

**Keywords:** CAH, Virilization, *CYP21A2*, 21-hyrdroxylase deficiency, *TNXA/TNXB* chimeric gene, Ehlers-Danlos syndrome, Contiguous gene syndrome, CAH-X syndrome

## Abstract

**Background:**

Genetic diversity of mutations in the *CYP21A2* gene is the main cause of the monogenic congenital adrenal hyperplasia (CAH) disorder. On chromosome 6p21.3, the *CYP21A2* gene is partially overlapped by the *TNXB* gene, the two residing in tandem with their highly homologous corresponding pseudogenes (*CYP21A1P* and *TNXA*), which leads to recurrent homologous recombination.

**Methods and results:**

In the present study, the genetic status of an ethnic Greek-Cypriot family, with a female neonate that was originally classified as male and manifested the salt-wasting (SW) form, is presented. Genetic defects in the *CYP21A2* and *TNXB* genes were investigated by Sanger sequencing multiplex ligation-dependent probe amplification (MLPA) and a real-time PCR assay. The neonate carried in compound heterozygosity the *TNXA/TNXB* chimeric gene complex (termed CAH-X CH-1) that results in a contiguous *CYP21A2* and *TNXB* deletion and in her second allele the pathogenic IVS2-13A/C > G (c.655A/C > G) in *CYP21A2*.

**Conclusions:**

The classic SW-CAH due to 21-hydroxylase (21-OH) deficiency may result from various complex etiological mechanisms and, as such, can involve the formation of monoallelic *TNXA/TNXB* chimeras found *in trans* with other *CYP21A2* pathogenic variants. This is a rare case of CAH due to 21-hydroxylase deficiency, which elucidates the role of the complex RCCX CNV structure in the development of the disease. Identification of the correct CAH genotypes for a given phenotype is of considerable value in assisting clinicians in prenatal diagnosis, appropriate treatment, and genetic counseling.

## Introduction

Congenital adrenal hyperplasia (CAH) is a group of autosomal recessive disorders that affect cortisol biosynthesis and cause defective steroidogenesis [1]. The most prevalent type of CAH is 21-hydroxylase (21-OH) (90–95% of cases), followed by the next most common type of 11β-hydroxylase (11β-OH) (∼ 5% of cases) and other infrequent types [2]. The disorder has a wide spectrum of clinical phenotypes, and severity depends on the patients’ underlying *CYP21A2* genotype [1, 3, 4]. Currently, based on the inherited genetic background, the disorder is classified into the “classic” and “non-classic” (NC late onset) CAH forms [1, 2]. In “classic CAH,” during the course of fetal development, the adrenal cannot sufficiently produce cortisol and overproduces androgens, leading to varying degrees of prenatal virilization of the external genitalia in affected girls [5]. The multiallelic and tandem RCCX copy number variation (CNV) locus is found on chromosome 6p23.1 within the major histocompatibility complex (MHC) class III region. The RCCX structure is characteristically defined by the copy number of a DNA segment containing a series of genes, namely, serine/threonine kinase 19 (*STK19*), complement 4 (*C4*), steroid 21-OH (*CYP21A2*), and tenascin-X (*TNX*). The most frequent RCCX haplotype (69%) comprises two segments that are situated close to each other, with the first one containing the pseudogenes *STK19-C4A-CYP21A1P-TNXA* and the second containing the genes *STK19B*-*C4B*-*CYP21A2*-*TNXB* [6]. The majority of deleterious variants, including about 75% of those reported in 21-OH deficiency cases, are transferred to the *CYP21A2* gene by small conversions from the *CYP21A1P* pseudogene during meiosis [6–8]. It is estimated that the remaining 20–30% of the common *CYP21A2* pathogenic variants in CAH cases involve large gene deletions or amplifications of the *CYP21A2* gene and the other contiguous genes. The formation of these mutants is the result of misalignments due to unequal crossing over during meiosis [6, 9–11]. To date, nine different haplotypes of chimeric *CYP21A1P*/*CYP21A2* genes (CH-1 to CH-9) in 21-OH deficiency have been detected [6, 12, 13]. Likewise, so far, three other chimeric recombination events between the *TNXB*/*TNXA *have been reported, which result in the deletion of the *CYP21A2* gene, thus creating a CAH disease-causing allele [14, 15]. These three types of *TNXB/TNXA* chimeras disrupt both the *CYP21A2* and the *TNXB* genes and, according to the type of disruption, have been termed CAH-X CH-1, CH-2, and CH-3 [15]. Approximately 10% of patients with CAH have CAH-X syndrome due to the presence of a monoallelic nonfunctional *TNXB/TNXA* chimeric gene in combination with another *CYP21A2* pathogenic mutation in the second allele [2]. The clinical phenotype of CAH-X includes joint hypermobility, arthralgias, joint dislocations, hernias, and cardiac malformations [2, 15]. The *TNXB* gene encodes for the large extracellular matrix glycoprotein tenascin-X (TNX) and has a significant structural function in the assembly of collagen deposition by dermal fibroblasts and the connective tissues of the heart and skeletal muscle [16]. Ehlers-Danlos syndrome (EDS), characterized by two autosomal recessive mutations in the *TNXB* gene, leads to complete deficiency of tenascin-X: patients typically have the classical hypermobile type of EDS, with a characteristically large range of joint movements [17, 18]. Interestingly, there have also been reports of heterozygous *TNXB* gene mutations causing tenascin-X haploinsufficiency, which also results in the signs and symptoms of hypermobile-type EDS [14, 19, 20].

In recent years, numerous studies have established a robust correlation between the genotype and the phenotype of CAH of a large number of *CYP21A2* defects [1–4, 21–27]. In this study, we present the genetic features of the disease in a family of Cypriot descent with the salt-wasting (SW) classic form as a result of the *TNXA/TNXB* chimera, resulting in deletions of *CYP21A2* and *TNXB* in one allele [7, 15] and a severe case of *CYP21A2* mutation in the second allele.

### Case description

#### Patients and bioethics approval

Written and oral informed consent was obtained from both parents of the infant under investigation screened for mutations in the *CYP21A2* gene. The study was approved by the Cyprus National Ethics Committee, and all methods performed were in accordance with the relevant guidelines and regulations.

#### History

A female neonate of non-consanguineous and healthy ethnic Greek-Cypriot parents was born at 40 weeks of gestation with normal delivery and an APGAR score of 10. On initial examination, the neonate was assigned as male based on the phenotypic appearance of the genitalia with an external genital appearance of Prader 5. After the first 2 weeks of life, the neonate was diagnosed with the salt-wasting (SW) form of CAH and reassigned as female based on biochemical findings of SW (failure to thrive, hyponatremia, hyperkalemia, high plasma renin activity (PRA), significantly high 17-OHP > 75.7 nmol/L, and high ACTH). Following the diagnosis of SW-CAH, the infant was started on replacement treatment with glucocorticoids and salt supplementation (hydrocortisone and fludrocortisone), with excellent response regarding linear growth and mental development. Salt supplementation was stopped at the age of 4 months, at which time the infant underwent surgery to correct the appearance of her genitalia where a vaginal opening was formed.

**Fig. 1:**
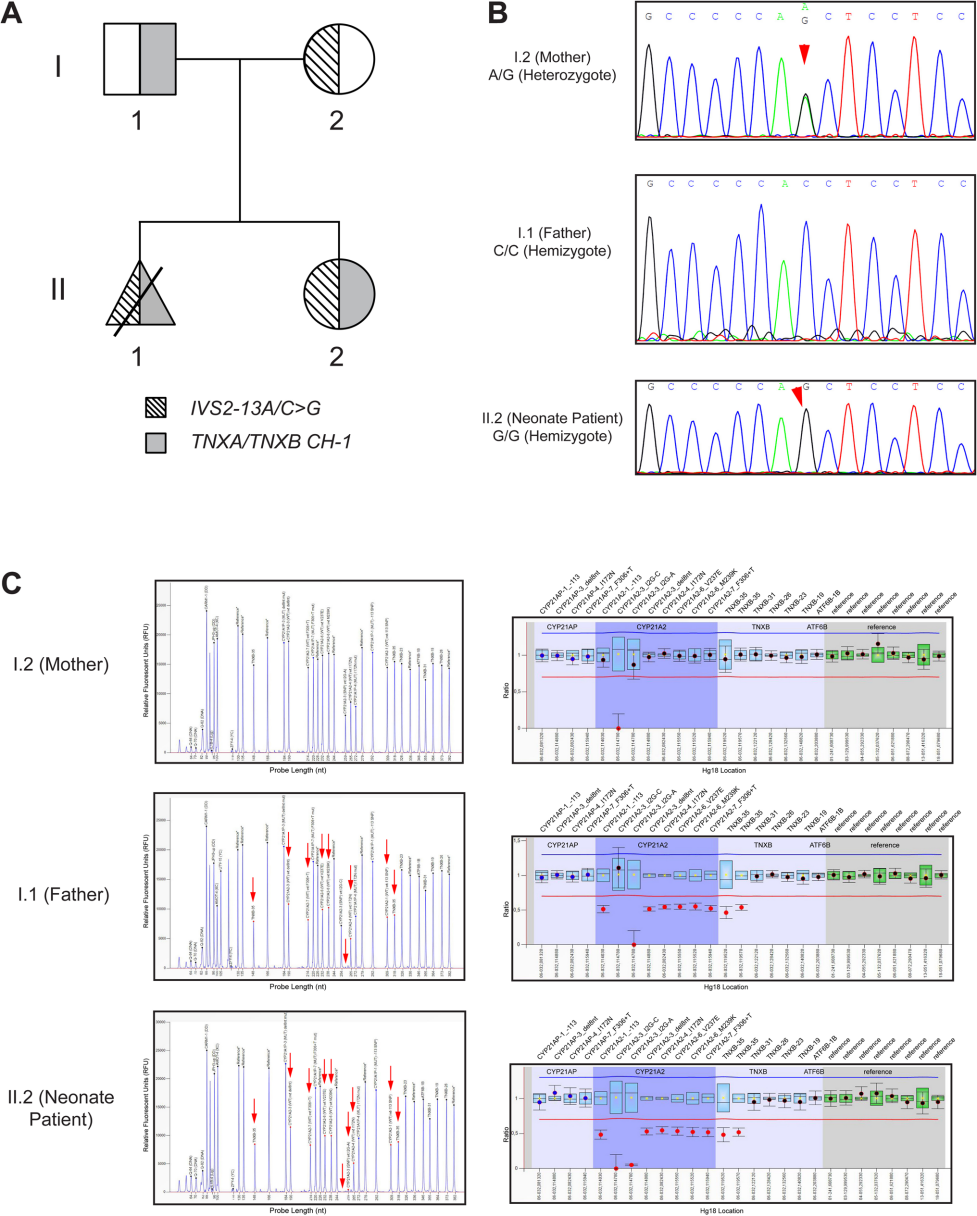
**A **Pedigree of the investigated family with congenital adrenal hyperplasia. Gray shading indicates the presence of the TNXA/TNXB CH-1 chimeric allele, and hatched lines indicate the presence of the IVS2-13A/C > G mutation. **B **Sanger sequencing electropherograms indicating the severe IVS2-13A/C > G mutation identified in heterozygosity in the mother and in hemizygosity in the neonate female patient. The position of the IVS2-13A/C > G mutation is shown by red arrows. **C** MLPA electropherograms (left) and ratio charts (right) showing the *CYP21A2* gene deletion extending to exon 35 of *TNXB*. The father and the neonate patient carried in heterozygosity the *TNXA/TNXB* CH-1 chimeric gene. Red arrows indicate the reduction of specific sites located on the *CYP21A2* and *TNXB* genes

A possible genetic diagnosis with the most severe SW classic form of CAH was speculated, and genetic investigation for mutations in the *CYP21A2* gene based on a cascade strategy, as formerly described, was undertaken for the infant and the parents [4, 28, 29]. The neonate was the first child born to this family, and no other close relative was reported with similar clinical issues. SW-CAH secondary to 21-OH deficiency in the infant was justified by the obtained Sanger sequencing and MLPA (MRC, Holland/SALSA MLPA Probemix P050 CAH) analyses (Fig. 1 pedigree and sequences, MLPA). The neonate was identified as carrying in compound heterozygosity the IVS2-13A/C > G (c.655A/C > G)/*TNXA/TNXB chimera* (termed CAH-X CH-1) and additionally proved to be a female and remained as such as evidenced by the MLPA analyses. The 30-year-old mother was identified as carrying in heterozygosity the pathogenic IVS2-13A/C > G, while the 28-year-old father was concurrently found to also carry in heterozygosity the *TNXA/TNXB* chimera (Fig. 1 pedigree and sequences, MLPA). The *TNXA/TNXB* chimera results from an infrequent breakpoint of the *TNXB* gene, leading to complete deletion of *C4B* and *CYP21A2* [15, 30].

**Fig. 2:**
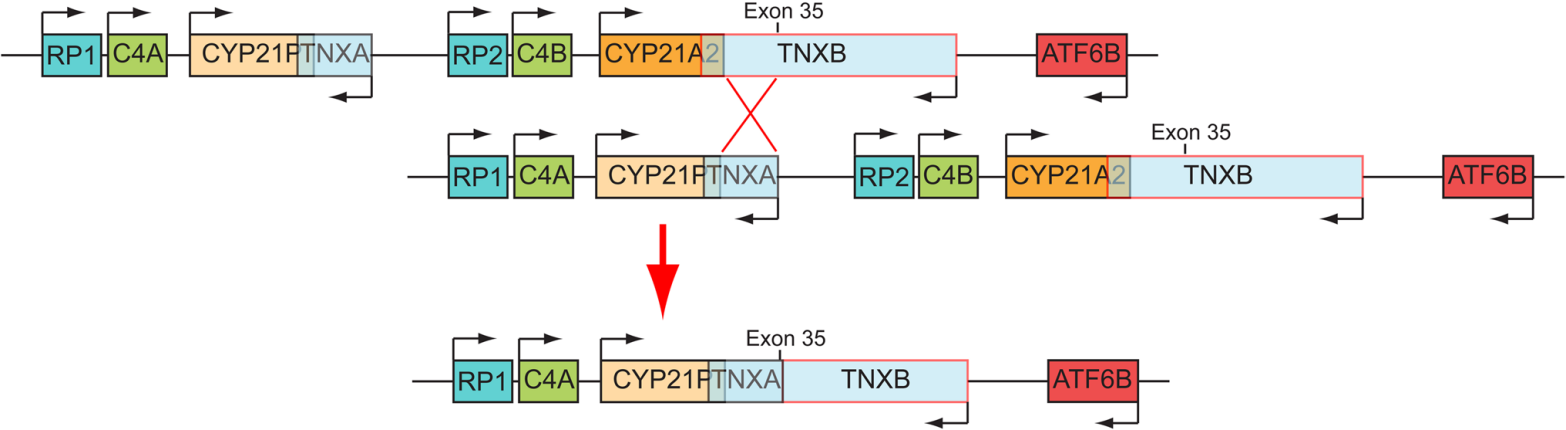
Schematic representation of the RCCX locus on chromosome 6p21.3 in which the *TNXA* and *TNXB* genes undergo unequal crossover resulting in a *TNXA/TNXB* CH-1 chimera

**Fig. 3:**
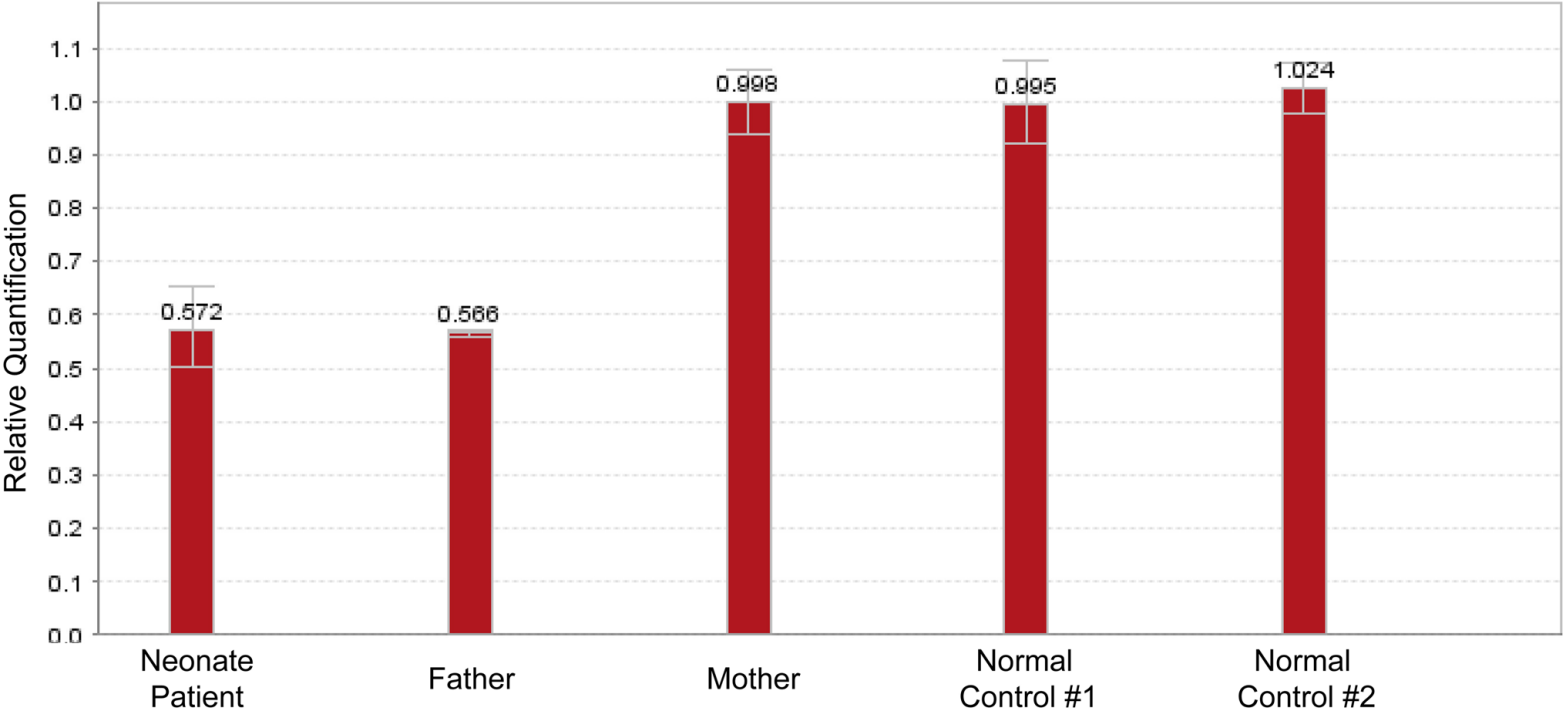
CAH Real Fast CNV Assay (real-time PCR, Vienna Lab) showing the large *CYP21A2* deletion that was detected both in the patient and in the paternal samples and which confirms the monoallelic *TNXA/TNXB* CH-1 chimera that was identified both in the father and in the neonate

This rare chimera is characterized by a 120-bp deletion in exon 35 of *TNXB* and results in *TNXB* haploinsufficiency and disrupted TGF-β signaling; when found in the homozygous state, it leads to a contiguous gene syndrome consisting of CAH and Ehlers-Danlos syndrome (EDS) [7, 31]. It has been reported that heterozygous *TNXB* mutations may be associated with the mild joint hypermobility form of EDS [1, 15] and that up to 10% of classic SW-CAH patients harboring in compound heterozygosity a *TNXB/CYP21A2* genotype tend to demonstrate an extended phenotype termed CAH-X syndrome [1, 31, 32] (Fig. 2). These patients have also been identified with clinical features of EDS, such as joint hypermobility, chronic arthralgia, hernias, and cardiac defects [7, 15]. The genetic investigation was extended by employing another test in addition to those suggested by the CAH Best Practice Guidelines [33]. Our investigation included the *CAH Real Fast CNV Assay (real-time PCR, Vienna Lab*) so as to re-confirm the large *CYP21A2* deletion that was detected both in the neonate and in the paternal samples. As demonstrated in Fig. 3, the large *CYP21A2* was likewise detected both in the neonate and in the paternal samples via the use of this method as well.

## Discussion

CAH-X syndrome is estimated to be found in approximately 7–9% of CAH patients [7, 14, 34] and is classified into three types (CAH-X CH-1, CAH-X CH-2, and CAH-X CH-3) as a result of chimeric recombination events between the *TNXB/TNXA *during meiosis, resulting in the deletion of the *CYP21A2* gene [15]. The CAH-X CH-1 type is the most frequent of the three and retains a 120-bp deletion at the boundary of exon 35 and intron 35 of *TNXB*. CAH-X CH-2 and CAH-X CH-3 types are less frequently found in CAH patients, with CAH-X CH-2 being characterized by the variant c.12174C > G (p.Cys4058Trp), which derives from the TNXA pseudogene [7], and CAH-X CH-3, being characterized by a cluster of three mutations (p.Arg4073His, p.Asp4172Asn, and p.Ser4175Asn) also derived from the *TNXA *pseudogene [35]. CAH-X CH-1 does not produce structural changes in the TNX protein but, rather, reduces the TNX expression, this being in contrast to the altered production of TNX proteins from both CAH-X CH-2 and CAH-X CH-3 [35]. An in-depth molecular genetic approach for the correct diagnosis of the severe forms of CAH, including CAH-X syndrome, is absolutely essential and indeed makes a difference to the everyday practice of an experienced endocrinologist. Lately, in addition to Sanger analysis, which still remains the gold standard for the detection of *CYP21A2* gene mutations, the PCR-based techniques of MLPA and real-time PCR assays are widely used for the detection of the severe deletions or duplications. Therefore, in the endocrinologist’s everyday practice, genetic information obtained by using these techniques aids towards taking the most appropriate decisions in cases of prenatal and preimplantation genetic diagnosis, hormonal treatment, and genetic counseling.

The female neonate with the classic SW-CAH of the present study presented the highest degree of virilization, with an external genital appearance of Prader 5, and, when salt supplementation was stopped at the age of 4 months, she underwent surgery for the correction of the appearance of her genitalia where a vaginal opening was formed.

Similarly to the neonate of the present study, CAH patients that carry in compound heterozygosity the splice IVS2-13A/C > G with the *TNXA/TNXB *(CAH-X CH-1) chimera generally have the most severe SW phenotype [36–38]. Several reports in European, Mediterranean, Middle Eastern, and Chinese populations confirmed a high frequency of the pathogenic IVS2-13A/C > G and found it to comprise 20 to 25% of the *CYP21A2 *mutant alleles and to translate to the most severe SW forms of the disease when inherited in homozygosity [1, 4, 27, 39–43]. Since the first CAH-X syndrome report by Merke et al. [14], it has been established that only 10% of CAH-X patients also have EDS manifestations [15, 44]. Several other studies that followed have reported an incidence of IVS2-13A/C > G combined with the chimeric *TNXA/TNXB* (CAH-X CH-1) genotype in patients with 21-OH deficiency and with no EDS manifestations [32, 44]. Similarly, the neonate of the present case with a compound heterozygous IVS2-13A/C > G/CAH-X CH-1 genotype presented only SW 21-hyroxylase deficiency and no EDS-related clinical manifestations. More specifically, our case has normal skin when compared to certain patients with biallelic CAH-X genotype and who typically demonstrate skin laxity and abnormal wound healing [45, 46]. The father of the neonate who was a carrier of the chimeric *TNXA/TNXB* (CAH-X CH-1) genotype also did not present any clinical manifestations of EDS.

In conclusion, we herein report an infrequent case of CAH illustrating that its clinical phenotype depends on the severity of the underlying variants, while the role of the complex RCCX CNV structure in the development of the disease is explained. Our report moreover constitutes an example of the complexities encountered in patients with classic CAH and adds to the understanding of the spectrum of CAH phenotype and the TNX-related disorders, including the CAH-X syndrome.
